# Transcriptomic and Hormone Analyses Provide Insight into the Regulation of Axillary Bud Outgrowth of *Eucommia ulmoides* Oliver

**DOI:** 10.3390/cimb45090462

**Published:** 2023-09-07

**Authors:** Ying Zhang, Dandan Du, Hongling Wei, Shengnan Xie, Xuchen Tian, Jing Yang, Siqiu Xiao, Zhonghua Tang, Dewen Li, Ying Liu

**Affiliations:** 1 College of Chemistry, Chemical Engineering and Resource Utilization, Northeast Forestry University, Harbin 150040, China; yingz_2ooo@163.com (Y.Z.); czy14747129511@139.com (D.D.); weily9033@126.com (H.W.); wode0512x@163.com (S.X.); 13803679486@163.com (X.T.); 18748120465@163.com (J.Y.); 18287386875@139.com (S.X.); tangzh@nefu.edu.cn (Z.T.); 2 Key Laboratory of Forest Plant Ecology, Ministry of Education, Northeast Forestry University, Harbin 150040, China; 3 Heilongjiang Provincial Key Laboratory of Ecological Utilization of Forestry-Based Active Substances, Harbin 150040, China

**Keywords:** axillary buds, *Eucommia ulmoides* Oliver, hormone, transcriptome

## Abstract

An essential indicator of *Eucommia ulmoides* Oliver (*E. ulmoides*) is the axillary bud; the growth and developmental capacity of axillary buds could be used to efficiently determine the structural integrity of branches and plant regeneration. We obtained axillary buds in different positions on the stem, including upper buds (CK), tip buds (T1), and bottom buds (T2), which provided optimal materials for the study of complicated regulatory networks that control bud germination. This study used transcriptomes to analyze the levels of gene expression in three different types of buds, and the results showed that 12,131 differentially expressed genes (DEGs) were discovered via the pairwise comparison of transcriptome data gathered from CK to T2, while the majority of DEGs (44.38%) were mainly found between CK and T1. These DEGs were closely related to plant hormone signal transduction and the amino acid biosynthesis pathway. We also determined changes in endogenous hormone contents during the process of bud germination. Interestingly, except for indole-3-acetic acid (IAA) content, which showed a significant upward trend (*p* < 0.05) in tip buds on day 4 compared with day 0, the other hormones showed no significant change during the process of germination. Then, the expression patterns of genes involved in IAA biosynthesis and signaling were examined through transcriptome analysis. Furthermore, the expression levels of genes related to IAA biosynthesis and signal transduction were upregulated in tip buds. Particularly, the expression of the IAA degradation gene *Gretchen Hagen 3* (*GH3.1*) was downregulated on day 4, which may support the concept that endogenous IAA promotes bud germination. Based on these data, we propose that IAA synthesis and signal transduction lead to morphological changes in tip buds during the germination process. On this basis, suggestions to improve the efficiency of the production and application of *E. ulmoides* are put forward to provide guidance for future research.

## 1. Introduction

*E. ulmoides* is the only species of the *Eucommiaceae* family [1]. *E. ulmoides* is one of the most extensively researched Chinese herbal medicines today [2] and has received much attention due to its medicinal and economic value [3,4]. It contains a variety of active compounds, such as chlorogenic acid, which is the main active ingredient in many Chinese medicinal herbs that has a definite effect on the treatment of hypertension [5]. As the utility and worth of *E. ulmoides* has been recognized, *E. ulmoides* has been cultivated and propagated in large numbers. Shoot branching is the main determinant of plant structure above ground, and it occurs through the growth of axillary shoot meristems called axillary buds [6]. It has been reported that shoot branches develop from axillary shoot meristems, which are established in the axils of each leaf base on the primary shoot axis and develop into lateral branches [7]. Branching has a prominent and fundamental contribution to the plant architecture [8] and determines the structural integrity of plant regeneration [9]. It is an important process in the development of plants, which depends on the growth of axillary buds [10]. Thus, the formation of axillary buds is a prerequisite and critical step for the initiation of branching. There are three phases to the development of an axillary bud: initiation in the axillary meristem in the leaf axil; the development of the axillary meristem; and the subsequent outgrowth or dormancy of the axillary buds [11]. Therefore, axillary bud development is advantageous to the sustainable and healthy development of *E. ulmoides* resources.

Plant hormones are a class of natural organic substances that play important roles in multiple physiological processes of plants at low concentrations (10^−6^ mol/dm^3^ or less) [12]. The major classes of plant hormones are auxins, cytokinins (CTK), gibberellins (GA), brassinolides, jasmonic acid, abscisic acid (ABA), ethylene, strigolactones, and salicylic acid [13]. It has been established that endogenous plant hormones regulate masses of physiological activities during the plant growth and development process [14]. Among them, indole-3-acetic acid (IAA), the most common naturally occurring active auxin [15], has been closely linked to the regulation of numerous aspects of plant growth and development, including the elongation and division of cells [16], vascular development [17], axillary bud germination [18], and the shoot architecture [19]. ABA is an important plant hormone that regulates plant development and resistance to biotic and abiotic stresses [20]. Kinetin (KT), a synthetic cytokinin plant hormone, belongs to the plant cytokinin family [21]. Zeatin (ZT) was the first naturally occurring cytokinin to be discovered [22], which can promote cell growth and regulate plant growth [23]. 6-Benzylaminopurine (6-BA), the first synthetic cytokinin, is widely used in plants to break dormancy [24]. As we all know, GA3 is the parent molecule of hundreds of gibberellins [25] and is the most important plant hormone in releasing dormancy and promoting germination [26]. Additionally, IAA plays a vital role in the regulation of flower bud growth [27] and bud elongation [28]. Moreover, some previous studies have demonstrated that plant hormones, such as CTK, GA, and ABA, play a pivotal role in regulating axillary bud growth [29,30,31,32].

Plant hormone signal transduction plays a very important role in hormone-related biochemical changes [33]. Changes in the expression of key genes in plant hormone anabolic pathways as well as hormone signaling pathways reflect endogenous plant hormone levels [34]. In the auxin signal transduction pathway, the main auxin-responsive genes include three gene families: auxin/IAA (Aux/IAA), Gretchen Hagen 3 (GH3), and small auxin up RNA (SAUR) [35]. AUX/IAA genes encode transcriptional repressors of auxin-responsive genes, while genes of the GH3 family regulate the auxin pool through negative feedback [36]. SAUR genes respond rapidly to auxin stimulation and can be transcribed by auxin within minutes without de novo protein synthesis [37]. In addition, the genes of the YUC family play an important role in IAA biosynthesis to maintain the auxin concentration and regulate plant growth [38].

More and more research has been carried out regarding transcriptomes in *E. ulmoides*. For example, the transcriptomes of female and male *E. ulmoides* flower buds were sequenced using the Illumina platform for the identification of genes related to floral development [39]. The transcriptome analysis elucidated the mechanism of phenylpropanoid and flavonoid regulation during the growth and development of *E. ulmoides* leaves [40]. Feng et al.’s [41] research demonstrated that several glycolytic genes may play crucial roles in α-linolenic acid accumulation in the kernels of *E. ulmoides*. However, research on the analysis of axillary bud germination via the transcriptome of *E. ulmoides* is relatively rare. In this study, the changes in hormone contents and the expression patterns of hormone-related genes were investigated. Based on our data and results, we suggested that enhancing the IAA content could improve the morphological transformation during bud germination, which could promote the proliferation efficiency of the bud to increase the *E. ulmoides* output. We expect that our results will provide a better understanding for the regulation of bud germination and development efficiency in *E. ulmoides*, which will be beneficial to future research on improving the reproductive efficiency of *E. ulmoides*.

## 2. Materials and Methods

### 2.1. Plant Materials and Growth Conditions

**Figure 1:**
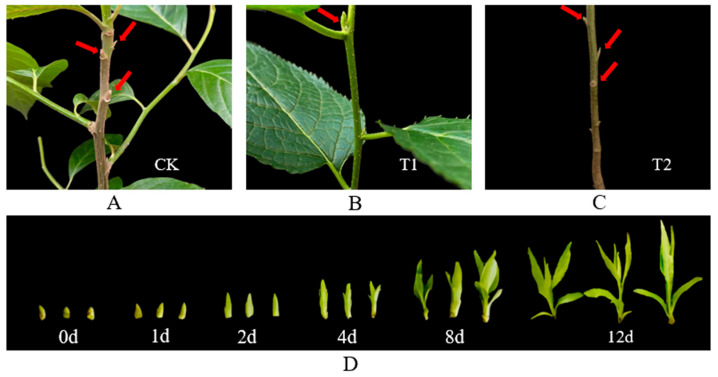
Morphological characterization of the three kinds of materials. (**A**) Phenotypes of upper buds (CK, red arrows). (**B**) Phenotypes of tip buds (T1, red arrows). (**C**) Phenotypes of bottom dormant buds (T2, red arrows). (**D**) Phenotypes from the shoot apex to the branches in T1.

Plants were cultivated in the greenhouse at Northeast Forestry University (NEFU) (45°43′ N, 126°38′ E), Harbin, Heilongjiang province in China. Three-year-old uniform-growth *E. ulmoides* seedlings were transplanted into 35 cm in diameter and 45 cm deep pots (one seedling per pot). The soil used for seedling growth was peat/vermiculite/perlite (1:1:1, *v*/*v*/*v*). Then, the tip buds (T1) and upper buds (CK) were sampled after day 0, day 1, day 2, day 4, day 8, and day 12 (Figure 1D). Three biological repeats for each sample were frozen in liquid nitrogen and immediately stored at −80 °C.

### 2.2. RNA Extraction, Library Preparation, and Transcriptome Sequencing

Total RNA was extracted using the TRIzol reagent (Sangon, Shanghai, China) according to the manufacturer’s instruction. The RNA quality was measured using NanoDrop 2000 (Thermo Scientific, Waltham, MA, USA), and Total RNA was reverse transcribed to cDNA using Biyuntian reverse transcription kit (Biyuntian, Shanghai, China) for library construction and then sequenced. Nine libraries, including three replicates of three bud samples, were created. Transcriptome sequencing was performed on an Illumina HiSeq platform (Illumina Inc., San Diego, CA, USA).

### 2.3. De Novo Assembly and Functional Annotation Analysis of Illumina Sequencing

First, short reads with a certain length of overlap were combined to form longer contigs. Then, clean reads were mapped back to the corresponding contigs based on their paired-end information. These contigs were then further processed with sequence clustering TGICL software (-F, version 2.1) to form longer sequences defined as unigenes. The generated unigenes were used for BLASTX alignment (E-value < 0.00001) against protein databases, including nonredundant, Swiss-Protein, Clusters of Orthologous Groups for Eukaryotic Complete Genomes, and Kyoto Encyclopedia of Genes and Genomes protein databases. With NR annotation, the Gene Ontology annotation and functional classification were performed using Blast2GO 2.5 and WEGO 2.0, respectively.

### 2.4. Identification of Differentially Expressed Genes (DEGs)

The level of gene expression was estimated by the value of the expected number of fragments per kilobase of transcript sequence per million base pairs sequenced (FPKM). Identification of differentially expressed genes (DEGs) was conducted using the program DESeq2 1.14.1. The resulting *p*-values were adjusted for controlling the false discovery rate. Genes according to the *p*-values < 0.05 and an absolute |log 2 (fold change)| ≥ 1.0 were identified as differential expression genes (DEGs). According to Zhang’s method, the DEGs were used for GO and KEGG enrichment analyses [42].

### 2.5. Measurements of Relevant Hormone Contents

*Eucommia ulmoides* Oliver (*E. ulmoides*) bud tissue was snipped, fully ground to a fine powder with liquid nitrogen, and transferred to a precooled 50 mL centrifuge tube that contained 3 mL of precooled 50% acetonitrile. Subsequently, the mixture was centrifuged at 10,000× *g* (10 min at 4 °C), and the supernatant was passed through a C18 extraction cartridge (Waters, Milford, MA, USA). The liquid was stored in a 50 mL centrifuge tube and taken to complete dryness in vacuo. Then, 1 mL of pre-cooled 30% acetonitrile was added to the tube to completely dissolve the hormone and filtered the samples through a 0.45 μm organic microfiltration membrane before loading. The samples were detected by high-performance liquid chromatography (HPLC) 1525 system (Waters, Milford, MA, USA).

### 2.6. Quantitative Real-Time PCR Validation

Nine genes were selected for validation using quantitative real-time PCR. Primer pairs were designed for qRT-PCR using Primer 5.0 (Thermo Fisher, Waltham, MA, USA). PCR reaction mixture contained 2 μL of diluted cDNA, 1.5 μL of reverse and forward primers, 5 μL of ddH_2_O, and 10 μL of the PCR master mix (Thermo Fisher Scientific, Waltham, MA, USA). Next, cDNA was amplified by ABI 7300 system according to the standard protocol, and the program was performed as follows: 95 °C for 2 min, followed by 40 cycles of 15 s at 95 °C, 30 s at 52 °C for and 60 s at 72 °C, 95 °C for 15 s, 60 °C for 15 s, 95 °C for 15 s, and 37 °C for 30 s. The amplification process was performed on the LightCycler^®^ 480II System (Roche, Basel, Switzerland; Roche Diagnostics, Indianapolis, IN, USA). The relative expression of the target gene was calculated based on 2^−ΔΔCt^ method [43] and using 40 s as the internal reference gene.

## 3. Results

### 3.1. Quality Assessment and Repeat Correlation Analysis of RNA-seq Data

To analyze the germination processes associated with the different position of the stem of axillary buds of *E. ulmoides*, nine cDNA libraries of *E. ulmoides* were sequenced, including CK (upper buds), T1 (tip buds), and T2 (bottom buds). There were three replicates per sample. A total of 71.10 Gb raw reads were generated, with raw reads count spanning from 21,090,187 to 42,863,152 and GC content between 46.39% and 48.21%. The highest average comparison efficiency of reference sequences of axillary buds of *E. ulmoides* was 81.73% (Table 1).

**Figure 2:**
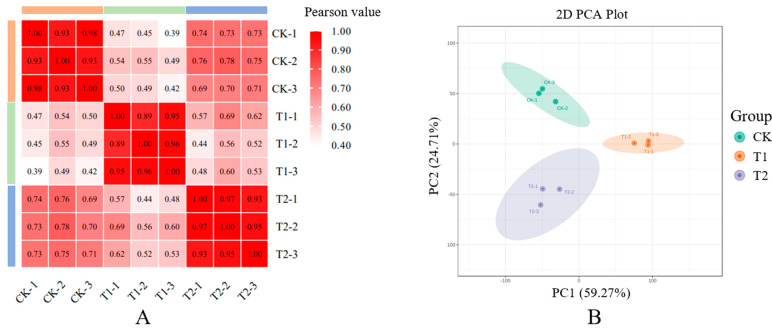
Pearson correlation coefficients of the sequencing data from three replicates of each sample collected from upper buds (CK), tip buds (T1), and bottom buds (T2) (**A**). Principal component analysis (PCA) of transcriptome data of the samples collected form CK, T1, and T2 (**B**).

The analysis of Pearson correlation demonstrated high correlations among the three replicates of each sample (Figure 2A). According to the three different kinds of axillary buds in *E. ulmoides*, the principal component analysis (PCA) divided samples into three groups (Figure 2B). Taken together, these results confirmed the high accuracy of transcriptome sequencing.

### 3.2. Analysis of DEGs in Different Comparison Groups

**Figure 3:**
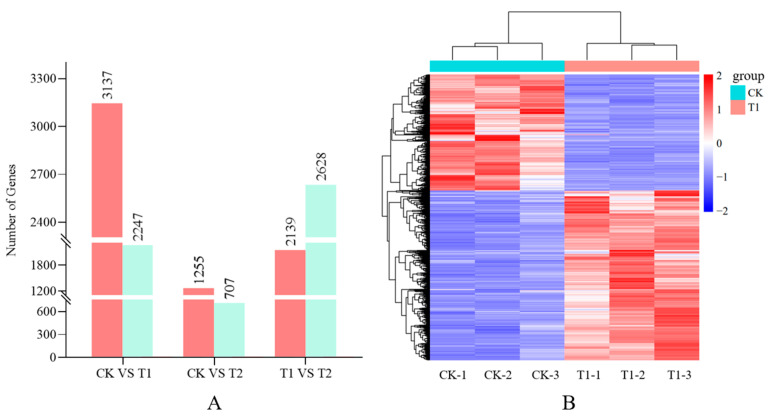
Analysis of differentially expressed genes (DEGs). (**A**) Statistical analysis of up- and downregulated DEGs between three groups. (**B**) Hierarchical cluster analysis of DEGs between CK and T1.

The CK vs. T1, CK vs. T2, and T1 vs. T2 comparisons of DEGs provided a clearer understanding of the up- and downregulation patterns between the three groups of samples. The comparisons revealed 3137, 1255 and 2139 upregulated genes and 2,247,707 and 2628 downregulated genes, respectively (Figure 3A). The number of DEGs in CK vs. T1 was significantly higher than the total number of DEGs in CK vs. T2 and T1 vs. T2, indicating that a large number of DEGs were involved in the germination of the tip bud pathway. This result indicated that the group of CK vs. T1 was the main comparison.

DEGs were analyzed by hierarchical clustering analysis of transcript abundances using the FPKM values to investigate the differences in gene expression trends between CK and T1, and these DEGs had different transcriptome profiles in the two axillary bud from different position of the stem (Figure 3B).

### 3.3. GO and KEGG Enrichment Analysis of DEGs

**Figure 4:**
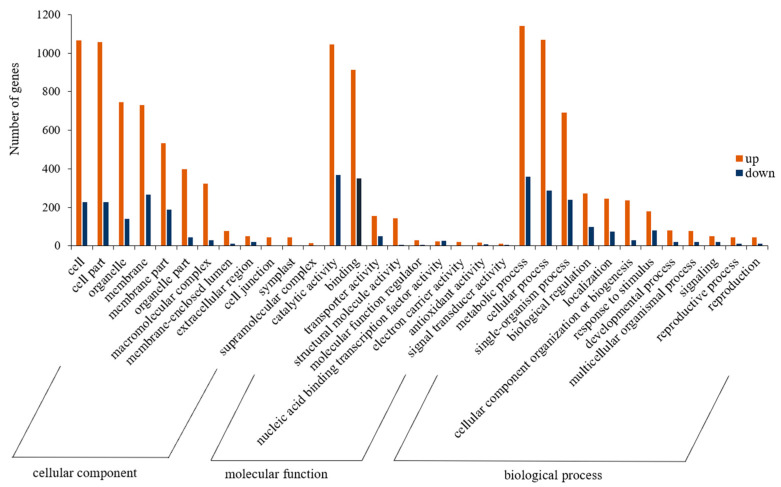
GO secondary classification of the set of DEGs between CK and T1.

GO analysis was performed on the set of DEGs identified between the CK and T1. The GO classification results showed that 6244, 3205, and 5400 unigenes were assigned to the GO categories of biological processes, cellular components, and molecular functions, respectively (Figure 4). In the classification of cellular components, upregulated DEGs were generally found in the cell, cell part, organelle, and membrane entries, while downregulated DEGs were focused on the membrane, cell, cell part, and membrane part. A substantial number of DEGs were linked to the catalytic, binding, and transporter activity in the classification of molecular function. In the classification of biological processes, up- and downregulated DEGs were mostly annotated to metabolic process, cellular process, and single-organism process entries, especially in the metabolic process category, which had the most concentrated DEGs.

**Figure 5:**
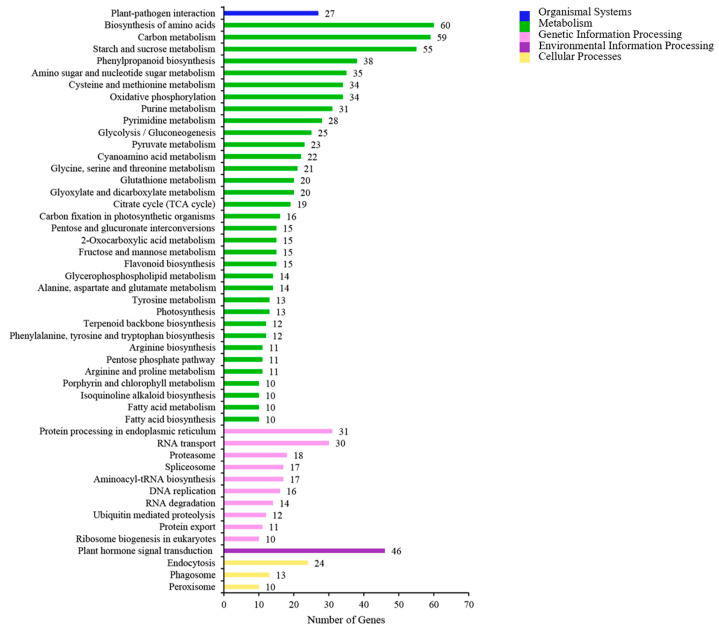
Secondary classification of the KEGG pathways of DEGs in CK vs. T1.

We also carried out KEGG pathway enrichment analysis on a group of DEGs screened via transcriptome sequencing for CK vs. T1 (Figure 5). The KEGG classifications were separated into cellular processes, environmental information processing, genetic information processing, metabolism, and organismal systems categories. In the cellular processes category, peroxisome (10 DEGs), phagosome (13 DEGs), and endocytosis (24 DEGs) were significantly enriched in CK vs. T1. Plant hormone signal transduction (46 DEGs) was significantly enriched in the environmental information processing category. Protein processing in the endoplasmic reticulum (31 DEGs) was significantly enriched in the genetic information processing category. The metabolism category had the largest number of DEGs, and these genes were focused on the biosynthesis of amnio acids (60 DEGs), carbon metabolism (59 DEGs), and starch and sucrose metabolism (55 DEGs) entries.

**Figure 6:**
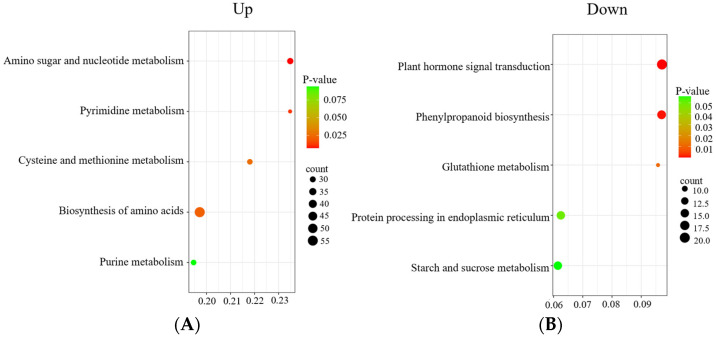
KEGG enrichment analysis of DEGs of CK vs. T1. (**A**) KEGG analysis of upregulated DEGs with CK vs. T1. (**B**) KEGG analysis of downregulated DEGs with CK vs. T1.

To gain more perspective into the enrichment of these DEGs in KEGG pathways, we performed separate KEGG enrichment analyses of DEGs from the group CK vs. T1 of *E. ulmoides*, as shown in Figure 5. The upregulated and downregulated DEGs were closely related to the biosynthesis of amino acids and plant hormone signal transduction pathway, respectively (Figure 6).

### 3.4. Validation of RNA-seq Data by RT‒qPCR

**Figure 7:**
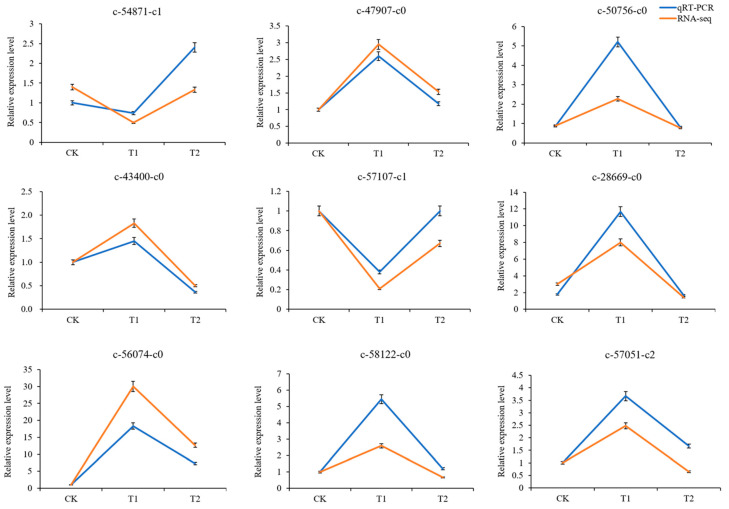
The comparison of the relative expression levels of the selected DEGs determined by RT-qPCR and RNA-seq.

We selected nine DEGs for RT‒qPCR validation in order to confirm the accuracy of the RNA-seq results. These DEGs were primarily chosen from genes that related to the auxin signaling pathway. As shown in Figure 7, the similarities between the RT‒qPCR results and RNA-seq data confirmed the accuracy of the RNA-seq results. Among these nine DEGs, the c-28669-c0 and c-56074-c0 showed the highest and second highest expression, respectively. The expression pattern of c-54871-c1 and c-57107-c1 differed from that of the other genes. The expression levels of most genes were higher in T1 than in CK and T2.

### 3.5. Quantification of Changes in Endogenous Plant Hormone Contents during Bud Development in E. ulmoides

**Figure 8:**
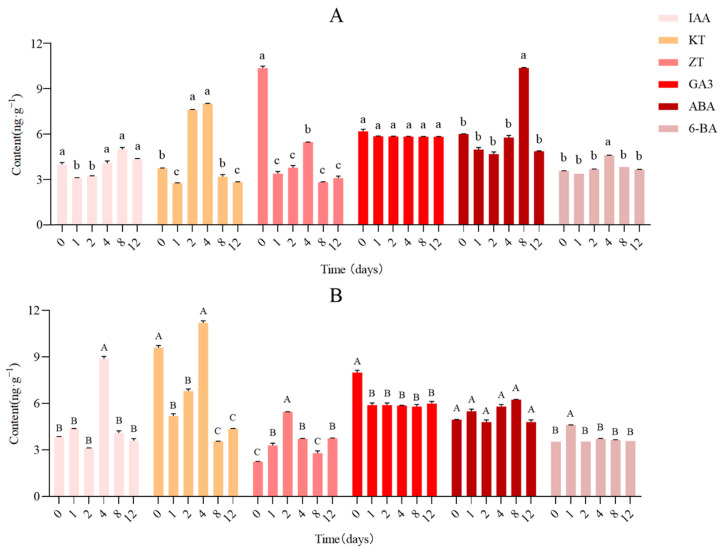
The levels of IAA, KT, ZT, GA3, ABA, and 6-BA in upper buds (CK) and tip buds (T1) of *E. ulmoides*. The contents of IAA, KT, ZT, GA3, ABA, and 6-BA in CK (**A**) and tip buds (**B**) of *E. ulmoides*. Values are presented as mean ± SE (*n* = 3), while different letters mean significant difference (*p* < 0.05).

We measured the content of GA3, IAA, KT, ABA, ZT, and 6-BA. There were significant differences among the contents of the six endogenous hormones (Figure 8). KT and IAA contents were higher than other hormones at day 4 in the tip buds, exhibiting a trend of first increasing (day 2 to day 4) and then decreasing (day 4 to day 8) (Figure 8B). The ZT content gradually increased during the early stage, demonstrating the diametrically opposite trend in upper buds (Figure 8A), and ZT content in the tip buds was higher than upper buds on day 2 and day 12 (Figure 8B). ABA content was the highest at day 8 in the upper buds, while ABA content was not dominant in the tip buds. There was no significant difference between the levels of GA3 and 6-BA in the upper and tip buds. Then, IAA content increased significantly (*p* < 0.05) in tip buds on day 4 compared with day 0, and was higher than upper buds on day 4.

### 3.6. The Integrated Analysis of DEGs Related to IAA Biosynthesis and Signaling Pathway in Tip Bud

**Figure 9:**
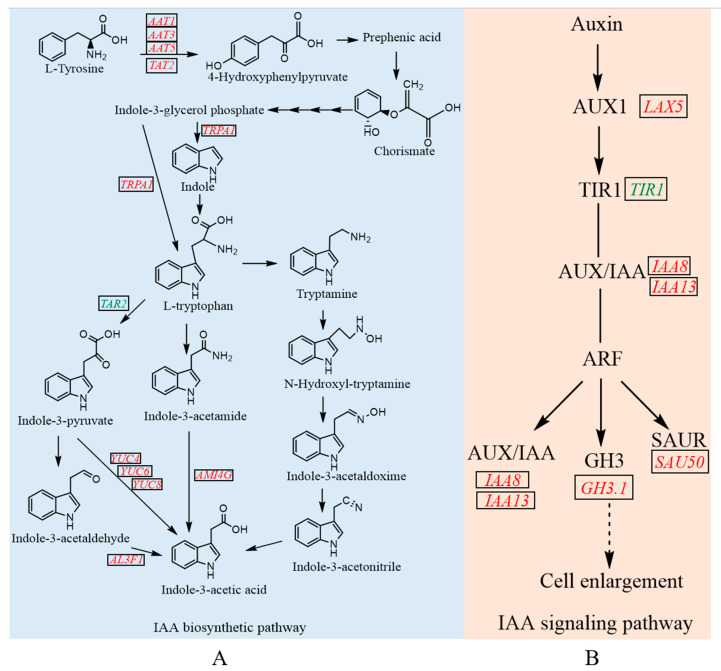
DEGs were involved in IAA biosynthesis pathway and signaling pathway in CK vs. T1 on day 4. (**A**) DEGs participated in IAA biosynthesis and metabolism pathway. (**B**) DEGs participated in IAA signaling pathway. The different font color represents the genes that are regulated in CK vs. T1 (red indicated upregulation; green indicated downregulation).

We identified a number of DEGs involved in endogenous hormone synthesis and signal transduction in CK vs. T1, including IAA, CTK, GA, ABA, brassinosteroid (BR), jasmonic acid (JA), and ethylene. Particularly, most of the DEGs were involved in IAA biosynthesis and the signaling pathway. In IAA biosynthesis and metabolism pathway, tyrosine aminotransferase [EC:2.6.1.5], aspartate aminotransferase, cytoplasmic [EC:2.6.1.1], histidinol-phosphate aminotransferase [EC:2.6.1.9], chorismate mutase [EC:5.4.99.5], tryptophan synthase alpha chain [EC:4.2.1.20], indole-3-pyruvate monooxygenase [EC:1.14.13.168], aldehyde dehydrogenase 3F1 [EC:1.2.1.3], and amidase [EC:3.5.1.4] were upregulated (Figure 9A). In the auxin signaling pathway, most of the genes encoding auxin influx carrier (AUX1), auxin-responsive protein IAA (AUX/IAA), indole-3-acetic acid-amido synthetase GH3 family, and SAUR family proteins were upregulated in the course of tip bud germination (Figure 9B).

### 3.7. Validation and Expression Analysis of Key Enzyme Genes

**Figure 10:**
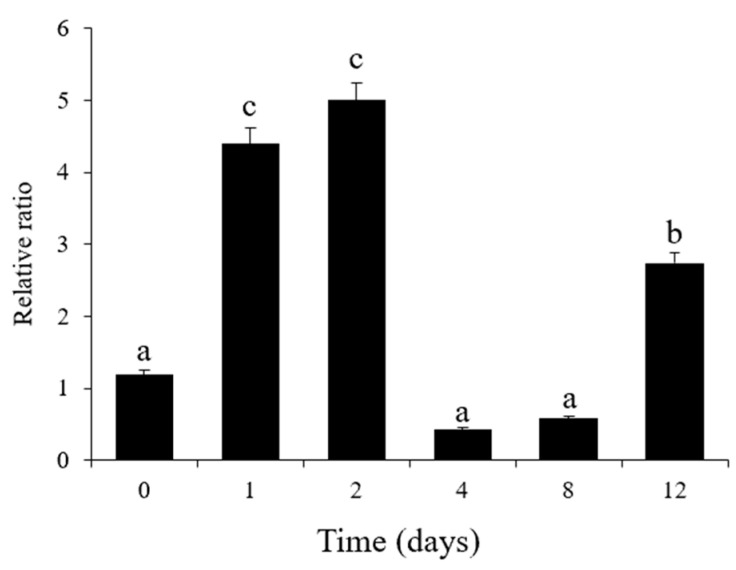
The expression of the *GH3.1* gene (this gene segment has 100% homology to *GH3.1* gene from the *Diospyros lotus*) was detected in tip buds (T1) (Ribosomal 40S protein S9 as an internal control) during different processes of germination. Values are presented as mean ± SE (*n* = 3), and different letters mean a significant difference (*p* < 0.05) with *n* = 3.

To analyze the expression profiles of the key enzyme gene involved in the IAA signaling pathway, the *GH3.1* gene (this gene segment has 100% homology to GH3.1 gene from the *Diospyros lotus*) was investigated by real-time quantitative PCR analysis (qRT-PCR) during different stages of the germination process. The results showed that the expression of the *GH3.1* gene (LOC127802819) was significantly (*p* < 0.05) upregulated from day 0 to day 2 (Figure 10), while the IAA content of tip buds (T1) was maintained in a low concentration range (Figure 8B). On day 4, the gene was significantly downregulated (Figure 10), but the content of IAA showed the opposite trend (Figure 8B). Then, the content of this gene gradually increased from day 8 to day 12 (Figure 10). However, the IAA content still showed the opposite trend at this stage and gradually decreased (Figure 8B).

## 4. Discussion

The germination process generally includes two developmental stages: the formation of leaf axil meristems and axillary bud outgrowth [44,45]. The increased number of lateral branches is caused by the axillary buds of *Arabidopsis* [46]. Auxin, a key plant hormone, regulates various cellular processes by altering the expression of diverse genes in plants [47]. Auxin is considered a systemic regulator, which plays an essential role in the regulation of the bud outgrowth process [48]. Previous studies reported that auxin synthesis and transport are essential for axillary meristem development and axillary bud growth [49,50]. In this study, IAA content significantly increased (*p* < 0.05) on day 4 compared with day 0 in upper axillary buds (Figure 8B), and the expression patterns of genes involved in IAA synthesis and metabolism were upregulated in tip buds to improve IAA concentration (Figure 9). In support of our findings, the content of IAA has gradually increased to promote the development of flower buds since the end of vernalization in Sorbonne [51]. Furthermore, young berries have the highest IAA level, which steadily decreases during the grape ripening process [52]. Moreover, the IAA content is higher in the early developmental stages and then declines throughout subsequent stages of berry development in the non-climacteric fruit [53]. However, in contrast herewith, the IAA content in tiller buds did not change significantly during the process of growth in wheat tillers [54]. Based on these results, we speculate that an increase in IAA content promotes bud germination and then maintains a stable level in the subsequent growth process.

IAA signaling is known to regulate the expression levels of early and primary auxin response genes through Auxin Response Factors (ARFs) [55], which can bind to auxin response DNA elements (AuxRE) of the genes to regulate plant growth and development [56]. The GH3 gene family participates in auxin conjugate formation and controls auxin-mediated signaling in plants [57]. It was shown that a group of auxin-inducible GH3 genes encode IAA-amido synthetase that regulates the endogenous IAA pool to reduce the concentration of free IAA through negative feedback [58]. A previous study demonstrated that IAA-amido synthase activity may explain the low levels of endogenous IAA in post-harvest papaya fruits [59]. In this finding, the changes in plant hormone contents were in agreement with the expression of genes related to plant hormone synthesis and signal transduction. And the expression of the gene *GH3.1* was significantly downregulated on day 4 (Figure 10), and the IAA content showed the exact opposite trend, with the highest on day 4 (Figure 8B). It was consistent with the finding that the auxin-responsive GH3 gene family reduced free IAA levels by binding excess IAA to amino acids [60]. Yuki Aoi et al., concluded that the GH3 auxin-amido synthetases can alter the levels of IAA in a GH3-dependent manner in *Arabidopsis* [61]. Moreover, an excess of auxin was conjugated with amino acids via the GH3 family of genes to maintain auxin homeostasis [62].

Regarding IAA synthesis pathways, plants mainly synthesize IAA from tryptophan via the indole pyruvate pathway, tryptamine pathway, indole acetaldoxime pathway, and indole acetamide pathway [63]. It has been reported that the tryptophan-dependent IAA synthesis pathway in plants is an important pathway for IAA biosynthesis [64]. In this study, the increased expression of genes involved in tryptophan metabolism corresponded to the increased IAA content observed in tip buds. The germination process of the buds requires the continuous support of endogenous IAA, and its content is higher in the germination stage, which may be due to the upregulation of the IAA synthesis of the YUC gene family and the downregulation of the IAA degradation genes *GH3.1*. On this basis, we preliminarily evaluated the effect of endogenous IAA content on bud germination and development in *E. ulmoides*, which provides a theoretical basis for the application of exogenous IAA to improve proliferation the efficiency of bud in production.

## 5. Conclusions

In this study, through the transcriptomic analysis of three different positions of axillary buds, it was found that a majority of DEGs (44.38%) were mainly found between CK (upper buds) and T1 (tip buds). It showed that DEGs were annotated in amino acid biosynthesis and plant hormone signal transduction pathways through the enrichment result of KEGG. We then analyzed the changes in exogenous hormone contents during bud initiation and development. Interestingly, the IAA content significantly increased (*p* < 0.05) in tip buds (T1) on day 4 compared with day 0 and showed much higher IAA content than upper buds (CK) on day 4. Furthermore, we analyzed the expression patterns of genes related to IAA biosynthesis and signal transduction through transcriptome analysis. Among them, the expression of IAA degradation gene *GH3.1* (this gene segment has 100% homology to GH3.1 gene from the *Diospyros lotus*) was downregulated on day 4, and IAA synthesis of the YUC gene family was upregulated in tip buds. Based on these findings, we found that the content of endogenous IAA showed an increase during bud germination, which was in agreement with the expression patterns of genes involved in IAA synthesis and signal transduction, demonstrating that increasing the IAA content was to the advantage of bud germination. Based on our study, we proposed that appropriately enhancing IAA content could improve the germination efficiency and proliferation efficiency of buds, which could be realized through the application of exogenous IAA concentration in the medium of buds in tissue culture production of *E. ulmoides.* All in all, our study provides a theoretical basis for the application of exogenous IAA to improve the bud proliferation efficiency of *E. ulmoides* in production.

## Figures and Tables

**Table 1:** A summary of the transcriptome sequencing data of the nine libraries constructed using corresponding samples at the three stages.

Sample	Raw Reads	Mapped Reads	Q30 (%)	GC Content (%)	Total Reads (%)	Mapped Reads (%)	Uniquely Mapped Reads (%)	Multiple Mapped Reads (%)
CK-1	21,090,187	16,622,007	93.13%	46.44%	100%	78.81%	31.39%	68.61%
CK-2	42,863,152	34,186,250	92.71%	46.39%	100%	79.76%	31.41%	68.59%
CK-3	19,543,667	15,452,823	93.02%	46.60%	100%	79.07%	31.73%	68.27%
T1-1	21,905,904	17,739,521	91.11%	46.46%	100%	80.98%	31.56%	68.44%
T1-2	29,965,914	24,595,359	93.04%	47.39%	100%	82.08%	31.96%	68.04%
T1-3	37,593,894	30,876,786	92.97%	47.12%	100%	82.13%	32.25%	67.75%
T2-1	21,323,173	16,947,499	91.49%	47.17%	100%	79.48%	30.62%	69.38%
T2-2	21,976,083	17,881,395	92.68%	48.21%	100%	81.37%	30.14%	69.86%
T2-3	21,443,839	17,003,703	91.71%	47.87%	100%	79.29%	30.40%	69.60%

## Data Availability

All data are contained within the article.
